# Functional Redundancy of Class I Phosphoinositide 3-Kinase (PI3K) Isoforms in Signaling Growth Factor-Mediated Human Neutrophil Survival

**DOI:** 10.1371/journal.pone.0045933

**Published:** 2012-09-24

**Authors:** Jatinder K. Juss, Richard P. Hayhoe, Charles E. Owen, Ian Bruce, Sarah R. Walmsley, Andrew S. Cowburn, Suhasini Kulkarni, Keith B. Boyle, Len Stephens, Phillip T. Hawkins, Edwin R. Chilvers, Alison M. Condliffe

**Affiliations:** 1 Department of Medicine, University of Cambridge School of Clinical Medicine, Addenbrooke’s and Papworth Hospitals, Cambridge, United Kingdom; 2 Inositide Laboratory, The Babraham Institute, Cambridge, United Kingdom; 3 Novartis, Horsham Research Centre, Horsham, United Kingdom; 4 Academic Unit of Respiratory Medicine, The Medical School, University of Sheffield, Sheffield, United Kingdom; University of Bern, Switzerland

## Abstract

We have investigated the contribution of individual phosphoinositide 3-kinase (PI3K) Class I isoforms to the regulation of neutrophil survival using (i) a panel of commercially available small molecule isoform-selective PI3K Class I inhibitors, (ii) novel inhibitors, which target single or multiple Class I isoforms (PI3Kα, PI3Kβ, PI3Kδ, and PI3Kγ), and (iii) transgenic mice lacking functional PI3K isoforms (p110δ^KO^γ^KO^ or p110γ^KO^). Our data suggest that there is considerable functional redundancy amongst Class I PI3Ks (both Class IA and Class IB) with regard to GM-CSF-mediated suppression of neutrophil apoptosis. Hence pharmacological inhibition of any 3 or more PI3K isoforms was required to block the GM-CSF survival response in human neutrophils, with inhibition of individual or any two isoforms having little or no effect. Likewise, isolated blood neutrophils derived from double knockout PI3K p110δ^KO^γ^KO^ mice underwent normal time-dependent constitutive apoptosis and displayed identical GM-CSF mediated survival to wild type cells, but were sensitized to pharmacological inhibition of the remaining PI3K isoforms. Surprisingly, the pro-survival neutrophil phenotype observed in patients with an acute exacerbation of chronic obstructive pulmonary disease (COPD) was resilient to inactivation of the PI3K pathway.

## Introduction

Neutrophils are terminally differentiated, short-lived innate immune cells, which contain an arsenal of cytotoxic agents essential for pathogen clearance. If activated inappropriately these microbicidal mechanisms can result in significant tissue injury [1]. Hence, neutrophil-mediated tissue damage plays a cardinal role in the pathogenesis and progression of several diseases, including acute respiratory distress syndrome (ARDS) [2], cystic fibrosis (CF) [3], and chronic obstructive pulmonary disease (COPD) [4]. Apoptosis controls neutrophil longevity in tissues and is critical to the resolution of granulocyte inflammation [5], [6]. Neutrophils undergo rapid constitutive apoptosis, and survival is contingent on the balance of pro-survival and pro-apoptotic signals derived from the micro-environment. The growth factor granulocyte-macrophage colony stimulating factor (GM-CSF) drives the aberrant neutrophil survival response observed in patients with ARDS and ventilator-associated pneumonia [5], [7], both common causes of death in Intensive Care Units. In animal models of lung inflammation, pharmacological acceleration of neutrophil apoptosis promotes the resolution of inflammation [6].

Class I PI3Ks play a critical role in transducing signals from cytokines, chemokines and growth factors by catalyzing the synthesis of key lipid-based second messengers, particularly phosphatidylinositol-3,4,5-trisphosphate (PtdIns(3,4,5)P_3_). This leads to engagement of downstream effectors such as PKB (Akt) and PDK1, which regulate fundamental cellular processes related to cell growth, proliferation, adhesion, migration and survival, reviewed in [8]. Structurally, Class I PI3Ks are heterodimers, comprising a 110 kDa catalytic subunit p110 (α, β, δ or γ) and an adaptor subunit (p55/p85 or p84/p101). Class I PI3Ks are subdivided into IA and IB; Class IA consist of p110α, β and δ, which associate with the p85 or p55 adaptor and are generally activated through receptor tyrosine kinases. By contrast, Class IB PI3Ks consists solely of p110γ, which associates with p101 or p84 adaptor subunits and is stimulated by the βγ subunits of G-protein coupled receptors. Class I PI3K heterodimers are conventionally named only by their catalytic subunit, thus PI3Ks α, β, δ or γ refer to dimers containing p110α, β, δ or γ, respectively.

There is evidence from multiple cells lines that individual Class I PI3K isoforms can play unique signaling roles in a variety of biological processes, reviewed in [9]. The p110δ and γ isoforms are enriched in immune cells (including neutrophils) [10] and are integral to leukocyte function. Mice lacking functional p110γ show impaired neutrophil and macrophage migration, reduced neutrophil oxidative burst activity, mast cell degranulation, and impaired B-cell and thymocyte development [11]–[14]. The PI3Kγ isoform has also been reported to exert significant anti-apoptotic effects in neutrophils even under basal conditions [15], [16]. PI3Kδ contributes to neutrophil chemotactic responses [17], their recruitment to inflammatory foci [18], and to the human (but not murine) neutrophil respiratory burst [10]. PI3Kβ has recently been shown to play a selective role downstream of neutrophil Fcγ receptors [19]. These PI3K isoforms consequently represent attractive therapeutic targets in inflammation and a number of inhibitors have already entered Phase I clinical trials.

Recently, Foukas et al. [20] demonstrated that signaling through any PI3K Class IA, but not class IB, could sustain survival in hemopoietic progenitor cells following treatment with IL-3. However, it is well established that immortalized cells exhibit dysregulated intracellular signaling and behave differently to primary cells [21]. Although multiple interdependent survival pathways co-exist in neutrophils, we have previously demonstrated that GM-CSF-mediated neutrophil survival is >85% PI3K-dependent [22]. Given recent evidence of PI3K isoform-specific roles, we wished to investigate the relative contribution of individual Class I PI3K isoforms to constitutive neutrophil apoptosis and the cytoprotective effect of GM-CSF. We used a panel of novel small molecule inhibitors and transgenic mice lacking one or more functional PI3K isoforms (p110δ^KO^γ^KO^ or p110γ^KO^). We report near-complete functional redundancy of the PI3K Class I isoforms in mediating the GM-CSF survival effect in both human and murine peripheral blood neutrophils, necessitating combined inhibition of at least three PI3K Class I isoforms to block the pro-survival effect of GM-CSF. With regard to the potential for modulation of neutrophil apoptosis in human disease states, we observed a clear neutrophil survival phenotype in patients experiencing an exacerbation of COPD, which was completely resistant to PI3K inhibition.

## Materials and Methods

### Ethics Statement

All human participants gave written informed consent, and all studies complied with the Declaration of Helsinki. Human peripheral blood neutrophils were purified from healthy human volunteers (ethical approval UK06/Q0108/281). Neutrophils were also isolated from the peripheral blood of patients admitted to hospital suffering from a non-infective acute exacerbation of COPD (ethical approval UK08/H0308/281). All animal studies were performed following approval by the UK Home Office Animals (Scientific Procedures) Act 1986 and in accord with the conditions specified in Project License PPL 80/2335.

### Mouse Strains

Mouse strains with deletions in the PI3Kγ (p110γ^KO^) or PI3Kγ and δ catalytic subunits (p110δ^KO^γ^KO^ ) were bred on a C57BL/6 background for at least 6 generations [14]. In all relevant experiments, neutrophils from these mutant strains of mice were compared with cells derived from appropriate strain-matched wild-type C57BL/6 controls. All mice were housed in the small animal barrier unit (SABU) at the Babraham Institute under specific pathogen-free conditions. Mouse husbandry and experimentation were conducted in accordance with UK Home Office Project License PPL 80/2335. Mice aged 12–16 weeks were terminally anesthetized using an intraperitoneal injection of fentanyl citrate (Vet Pharma) and midazolam (Roche).

### Isolation of Human Peripheral Blood Neutrophils from Healthy Volunteers and Patients with COPD

Human peripheral blood neutrophils were purified from healthy human volunteers (ethical approval UK06/Q0108/281) by dextran sedimentation and centrifugation through plasma/Percoll gradients. Cell purity was consistently greater than 97% neutrophils as assessed using cytocentrifuge preparations fixed in methanol and stained with DiffQuick™. Neutrophils were also isolated from the peripheral blood of patients admitted to hospital suffering from a non-infective acute exacerbation of COPD (ethical approval UK08/H0308/281) with written informed consent. Details of patients with COPD are given in Table 1. All patients recruited had COPD defined according to GOLD criteria stage 2–3 as judged by post-bronchodilator spirometry when stable, and an exacerbation requiring hospital admission. Patients taking long term oral corticosteroids or those who had been treated in the community for more than 48 hours prior to admission were excluded, as were those with consolidation on a chest radiograph, an elevated C-reactive protein level, or sputum cultures positive for bacterial pathogens. All patients received nebulised bronchodilators and oral corticosteroids from the time of admission; other treatment was at the discretion of the attending physician. None of the patients studied required mechanical ventilation. Patients were venesected within 48 hours following hospitalization and a further sample obtained following discharge within 14 days of admission; by this point all patients were judged to have been clinically stable for at least 5 days. Of the patients recruited, one self-discharged from hospital within 5 days of admission and did not complete the study.

**Table 1:** Demographic profile of the patients with chronic obstructive pulmonary disease (COPD).

COPD Patient	Age (yrs)	Smoking status	FEV1/FVC ratio (%)	CXR	WBC (×10^9^/L)	CRP (mg/L)
1	50	Current	60.3	Hyperinflated	10.1	3
2	80	Current	30.6	Cardiomegaly	12.3	6
3	70	Ex-smoker	57.1	Normal	15.5	6
4	68	Ex-smoker	63.5	Cardiomegaly	10.9	8
5	63	Current	38.9	Hyperinflated	8.1	6

**Figure 1:**
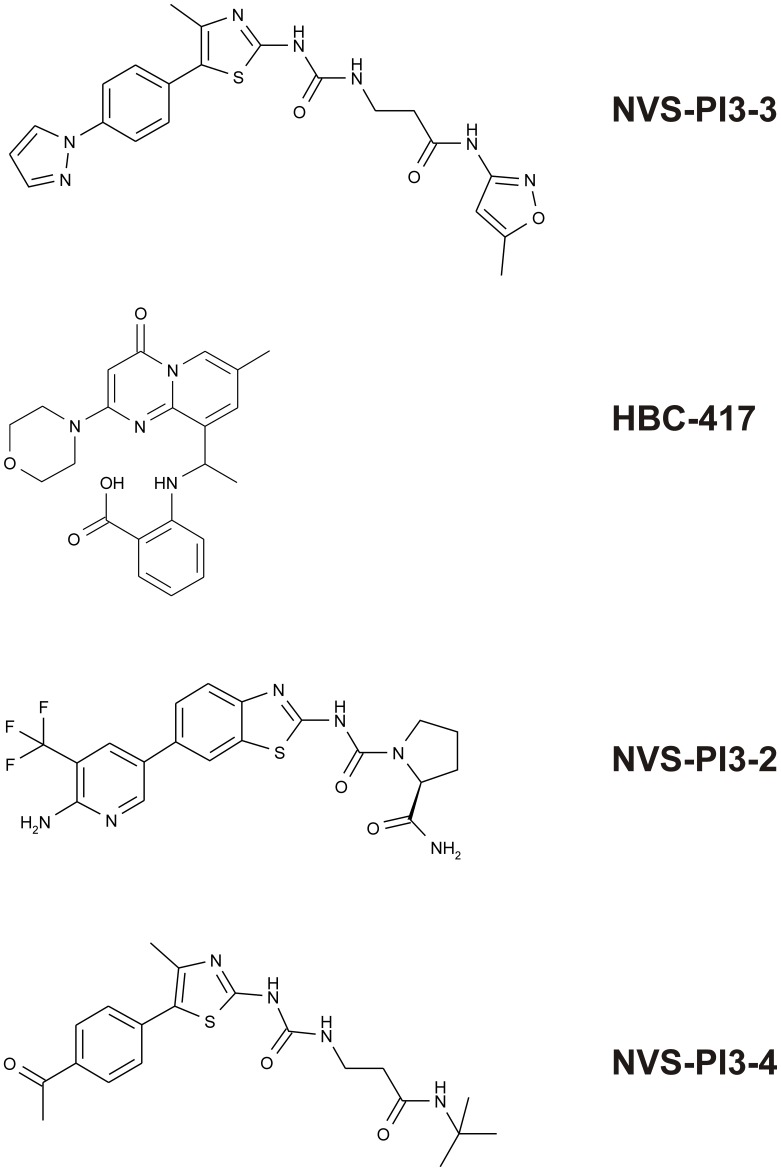
Molecular structures of NVS-PI3-2 (α inhibitor), HBC-417 (β inhibitor), NVS-PI3-3 (δ inhibitor) and NVS-PI3-4 (γ inhibitor).

To examine the effects of small molecule PI3K inhibitors on rhGM-CSF (10 ng/ml) -modulated apoptosis, neutrophils (5×10^6^/ml) were re-suspended in Iscove’s modified Dulbecco medium (IMDM) supplemented with 10% autologous serum, 100 U/mL penicillin and 100 µg/mL streptomycin in non-tissue culture treated flat bottom Falcon Flexiwell plates in a humidified 5% CO_2_ atmosphere at 37°C. Cells were pre-incubated for 20 minutes with DMSO vehicle control, LY294002 (10 µM); wortmannin (1 µM), PI-103(10 µM), YM-024 (3 µM), TGX-221 (10 µM), IC871184 (3 µM) or AS605240 (10 µM), or 1 µM of each of the Novartis PI3K inhibitors NVS-PI3-3, HBC-417, NVS-PI3-2 and NVS-PI3-4 (chemical structure, Figure 1) prior to treatment with rhGM-CSF (10 ng/ml). Wortmannin was replenished at 4, 8 and 12 hours to overcome its lack of stability in aqueous solution.

### Isolation of Murine Peripheral Blood Neutrophils

Studies were performed in accordance with the Project License PPL 80/2335. Blood (800 µl ±100 µl) was collected from the inferior vena cava of terminally anesthetized mice using a heparinized syringe; 100 µl was anti-coagulated with EDTA to perform peripheral blood counts by blood films and by using the Hematology Cell Counter MS9-5 (Mellet Schloesing Laboratories, France) (Table 2).

**Table 2:** Murine full blood counts with differential leukocyte count.

Parameter	C57BL/6	PI3Kγ^−/−^	PI3K δ^D910A^γ^KO^
Total WBC (x10^6^/mm^3^)	7.15±0.59	6.82±1.4	6.01±0.94
Lymphocytes (%)	74.1±0.14	54.8±5.21	55.8±3.00
Monocytes (%)	12.1±0.14	7.20±1.42	9.40±0.60
Neutrophils (%)	11.5±0.71	25.6±2.29	23.2±1.57
Eosinophils (%)	0.75±0.35	8.91±1.73	11.1±1.59
Basophils (%)	0.50±0.50	0.62±0.58	0.53±0.71
Haemoglobin (g/dl)	14.0±0.70	13.8±2.61	13.3±1.72
MCV (fl)	50.9±0.03	51.6±0.57	52.4±0.71
Platelets (x10^6^/mm^3^)	862±36.0	954±246	1034±135.0

Peripheral blood cell counts for 12 week-old C57BL/6 (n = 12), p110γ^KO^ (n = 4) and p110δ^KO^γ^KO^ (n = 3) mice on C57BL/6 background using an automated Hematology Cell Counter. Similar results were obtained by blood films (data not shown). Values are given as the mean ± SD. WBC, white blood cells.

The remaining blood was transferred into dextran (3 ml, 1.25% w/v in saline) as previously described [23]. The tubes were topped up to 10 ml with dextran solution and the erythrocytes sedimented at room temperature for 30 minutes. The leukocyte-rich supernatant was washed in buffer (sterile-filtered PBS without cations, containing 0.3% w/v low-endotoxin BSA, pH 7.4). Neutrophils were then negatively selected by incubating with the following antibodies: for lymphocytes, anti-CD2 (BD Biosciences 553109), -CD5 (BD Biosciences 553017) and -CD45R (BD Biosciences 553083), and for monocytes anti-F4/80 (Serotec MCAP497) and CD 115 (Serotec MCA1898XZ) as described, [23]. The final neutrophil yield was ∼0.7×10^6^ cells from each mouse. Cell purity was assessed by differential counts of cytocentrifuge preparations and by flow cytometry; samples of 90±5% purity were obtained for all reported experiments. Viability of freshly isolated neutrophils was consistently above 97% as assessed by both trypan blue staining and flow cytometry with propidium iodide.

Neutrophils were cultured at 1.0×10^6^/ml in Roswell Park Memorial Institute medium-1640 (RPMI), 10% heat inactivated fetal bovine serum (FBS) with 20 mM HEPES, and penicillin and streptomycin in the presence of various treatments in Flexiwell plates at 37°C in a 5% CO_2_ atmosphere.

### Morphologic Analysis of Neutrophil Apoptosis

Neutrophils were harvested from the 96-well at the time points indicated; human neutrophils were cytocentrifuged for 3 minutes at 300 rpm at high acceleration (murine neutrophils for 6 minutes at 900 rpm and low acceleration) onto Polysine™ microscope slides using a Shandon Cytospin 3 (Shandon, UK) then fixed in methanol and stained with DiffQuick™. Neutrophil apoptosis was assessed by cell morphology under light microscopy using a ×40 or ×100 objective. Apoptotic neutrophils were identified by cell shrinkage, blebbing of the cell membrane and the presence of darkly stained, condensed and fragmented nuclei. For each condition, triplicate slides were prepared and 300 neutrophils counted per slide in a blinded fashion. Brightfield images of the cytocentrifuge preparations were captured through the ×100 oil immersion objective SPlanApo 1.4 NA on the Olympus BX41 light microscope using Micropublisher 3.3 (Qimaging) camera and Q Capture-Pro (Qimaging) software.

### Flow Cytometry Analysis of Neutrophil Apoptosis

Neutrophils were harvested at the time points indicated and centrifuged (275 g, 1 minute) then re-suspended in 200 µl HEPES (N-2-hydroxyethylpiperazine-N’-2-ethanesulfonic acid) buffer (10 mM HEPES-NaOH, pH 7.4, 150 mM NaCl, 5 mM KCl, 1 mM MgCl_2_, 1.8 mM CaCl_2_) containing annexin-V-fluorescein isothiocyanate (FITC) (1 µg/ml) and propidium iodide (10 µg/ml). Samples were incubated for 20 minutes at 4°C in the dark, and the volume increased to 500 µl with HEPES buffer immediately before analysis using a fluorescence-activated cell sorter (FAC Calibur; Becton Dickinson, UK) with CellQuest software. Ten thousand events were collected for each sample, and data analyzed using FlowJo6.4.7 software. Viable, non-apoptotic neutrophils were defined as negative for both annexin V-FITC and PI, and apoptotic neutrophils were defined as positive for annexin V-FITC. Apoptosis was expressed as a percentage of apoptotic neutrophils in relation to the total number of counted neutrophils.

### Confocal Imaging

Freshly isolated neutrophils were incubated in the dark at 37°C either with the cationic mitochondrial dye 5, 5′, 6, 6′-tetrachloro-1,1′,3,3′-etraethylbenzimidazocarbocyaniniodide (JC-1, 10 µg/ml), or with annexin V-FITC and propidium iodide as described above, prior to imaging. Live-cell confocal images of murine peripheral blood neutrophils were obtained using cells cultured on sterile poly-lysine coated glass cover slips at 1.0×10^6^/ml in Roswell Park Memorial Institute medium-1640 (RPMI), 10% heat inactivated fetal bovine serum (FBS) with 20 mM HEPES at 37°C. Images were captured using an Olympus CellR imaging system comprising an Olympus IX81 microscope, Olympus MT-20 illumination system, Olympus ×100 SPlanApo ×100 1.45 NA objective, Hamamatsu Orca ER camera, and Olympus SIS software. For alexafluor 488-labelled annexin and PI, red/green images were taken every 10 minutes over 12 hours using a 300 ms exposure time and excitation intensity at 4% for both fluorescence channels; for JC-1, red/green images were taken using a 250 ms exposure time and excitation intensity set to 3% for both fluorescence channels. For both annexin/PI and JC-1 experiments transmitted light images were automatically captured in sequence with the fluorescence images using standard differential interference contrast optics.

### Western Blot Analysis for Akt Phosphorylation

Neutrophils were suspended at 5×10^6^/ml and 0.5 ml aliquots were pre-incubated for 20 minutes with PI3K inhibitors at 37°C prior to treatment with GM-CSF 10 ng/ml for 10 min. The cells were washed with ice-cold phosphate-buffered saline and pelleted and resuspended in 50 µl of cold lysis buffer (20 mM Tris-HCl (pH 7.5), 150 mM NaCl, 1 mM EDTA, 1 mM EGTA, 1% Triton X-100, 2.5 mM sodium pyrophosphate, 1 mM β-glycerophosphate, 1 mM PMSF, 1 mM sodium fluoride, 2 mM sodium orthovanadate, 10 µg/ml leupeptin, 10 µg/ml aprotinin) and incubated on ice for 10 minutes. The Triton-insoluble fraction was pelleted by centrifugation at 10 000 g for 15 min at 4°C. The protein concentration of the supernatant was determined using a colorimetric assay (bicinchoninic acid; BCA, BIO Rad Laboratories). SDS sample buffer was added to each neutrophil lysate and heated to 99°C for 5 min. 30 µg of protein/well was loaded on to each 10% SDS-polyacrylamide gel and subjected to electrophoresis at 150 V and transferred to a PVDF Immobilon membrane using a semi-dry transfer system at 130 mA for 90 minutes. The membrane was blocked for 1 hour with 5% non-fat dry milk in Tris-HCl buffered saline containing 0.1% (w/v) Tween 20 then immunoblotted overnight at 4°C in TBS-Tween-20 with primary monoclonal antibody to total Akt rabbit mAb or Phospho-Akt (Ser^473^) rabbit mAb (Cell Signaling Technology 40585 and 9272 respectively) at a dilution of 1∶1000 in TBS-Tween 20 with 5% Bovine Serum Albumin. The membrane was washed three times in TBS-Tween 20 and incubated with ECL HRP-conjugated donkey anti-rabbit IgG (Fab_2_ fragment; GE Healthcare) at a dilution of 1∶10 000 in TBS-Tween 20 for 1 hour at room temperature. Membranes were developed using ECL-Plus kit (GE Healthcare).

### Small Molecule Inhibitors and Cytokines

**Figure 2:**
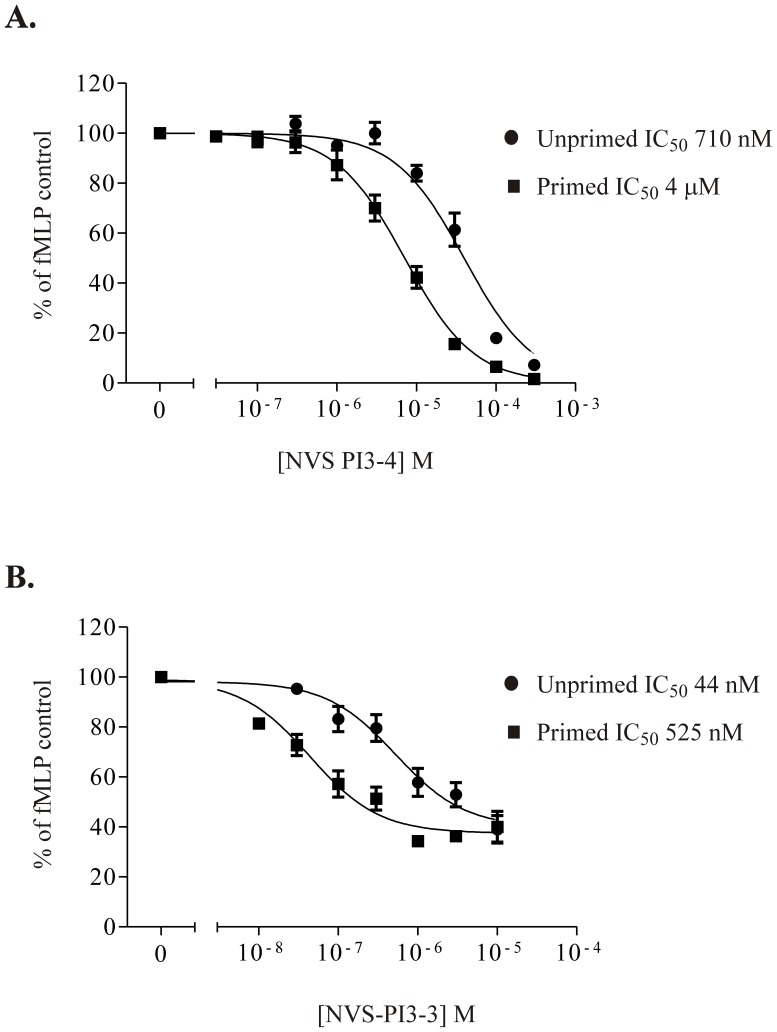
Effect of NVS-PI3-4 and NVS-PI3-3 on Neutrophil Oxidative Burst. Human neutrophils were suspended at 4×10^6^/ml in HBSS at 37°C and either primed with TNFα 10 ng/ml or treated with vehicle control, prior to the addition of NVS-PI3-4 (A) or NVS-PI3-3 (B) at the indicated concentrations. Luminol and HRP (1 µM and 62.5 U/ml final concentrations respectively) were added and aliquots (150 µl) transferred to a pre-warmed 96-well luminometer plate. fMLP (100 nM final concentration) was added via the injection port of a CentroPhago luminometer (Berthold Technologies, Hertfordshire, UK) and light emission was recorded over 5 min. Triangular symbols represent TNFα-primed cells; unprimed cells depicted by squares.

All PI3K inhibitors were dissolved in dimethyl sulfoxide (DMSO) and stored at −20°C. Recombinant human (rh) GM-CSF was obtained from R&D Systems, recombinant mouse (rm) GM-CSF was purchased from Peprotech and RU-486, and AS605240 were purchased from Sigma. The broad spectrum PI3K inhibitors LY294002 and wortmannin were obtained from Calbiochem and Sigma respectively; the pan PI3K Class I inhibitor PI-103 was purchased from Caymen Chemicals, Class IA PI3K isoform–selective inhibitors were as previously described [10]. The following compounds were provided by Novartis, NVS-PI3-3 (p110δ selective), HBC 417 (p110β selective), NVS-PI3-2 (p110α selective) and NVS-PI3-4 (p110γ selective). The IC_50_ values for each isoform-selective PI3K inhibitor (*in vitro* kinase assays) are listed in Table 3. The structures of NVS-PI3-2-4 and HBC-417 are given in Figure 1. HBC-417 is now commercially available as HY-75124 from Chemexpress. The concentration response curves for NVS-PI3-3 and NVS-PI3-4 on the neutrophil respiratory burst generated as described in [10] are shown in Figure 2.

**Table 3:** In vitro IC_50_ (µM) of small molecule phosphoinositide 3-kinase inhibitors.

PI3K inhibitor	p110α	p110β	p110δ	p110γ
LY294002	0.70	0.306	1.33	7.26
Wortmannin	0.001	0.01	0.005	0.009
PI-103 (pan class I)*	0.0008	0.088	0.048	0.15
YM-024 (α and δ)	0.3	2.65	0.33	9.07
TGX-221 (β)	5	0.007	0.10	3.5
IC87114 (δ)	>100	75	0.50	29
AS605240 (γ)*	0.06	0.27	0.3	0.008
NVS-PI3-2 (α)	0.075	5.5	0.98	2.4
HBC-417 (β)*	0.38	0.007	0.03	0.2
NVS-PI3-3 (δ)	0.180	0.60	0.003	0.31
NV3-PI3-4 (γ)	1.8	0.25	0.75	0.09

Data compiled from published work: LY294002, wortmannin, PI-103, YM-024, TGX-221, IC87114, AS605240.

Data generated by scintillation proximity assay performed in a final volume of 50 µL per well using a final concentration of ATP and PI in the assay of 5 µM and 6 µg/mL respectively: NVS-PI3-2, HBC-417, NVS-PI3-3, NVS-PI3-4.

* Poor cell accessibility.

### Antibodies, Reagents and Equipment

Rat anti-mouse antibody to CD2 (clone RM2-5), CD5 (clone 53-7.3), and CD45R (clone RA3-6B2) were purchased from BD PharMingen (Oxford, UK). Rat anti-mouse F4/80 Ag (Clone A3-1) and CD115 (clone 604B5 2E11) were purchased from Serotec (Kidlington, U.K.). Goat anti-rat IgG microbeads, quadroMACS® separator and LD columns were obtained from Miltenyi Biotec (Bisley, UK). Primary monoclonal antibody to total Akt rabbit mAb and Phospho-Akt (Ser^473^) rabbit mAb were obtained from Cell Signaling Technology. Annexin V-FITC and propidium iodide were purchased from BD Bioscience. Heat inactivated Fetal Bovine Serum (FBS) was purchased from Bioclear (Wiltshire, UK). Roswell Park Memorial Institute medium-1640 (RPMI) was obtained from Invitrogen (Auckland, NZ). All other reagents were as previously described [22].

### Statistical Analysis

Results were analyzed using Predictive Analytics SoftWare (PASW) Statistics 18.0.2 software and expressed as mean ± SEM of (n) separate experiments. Analysis of variance (ANOVA) analysis followed by Tukey HSD (Honestly Significant Difference) post-hoc tests was used to compare data and generate *p* values, *p*≤0.05 was considered significant.

## Results

### Inhibition of Multiple Class I PI3K Isoforms is Necessary to Abrogate the rhGM-CSF Cytoprotective Effect in Human Neutrophils

**Figure 3:**
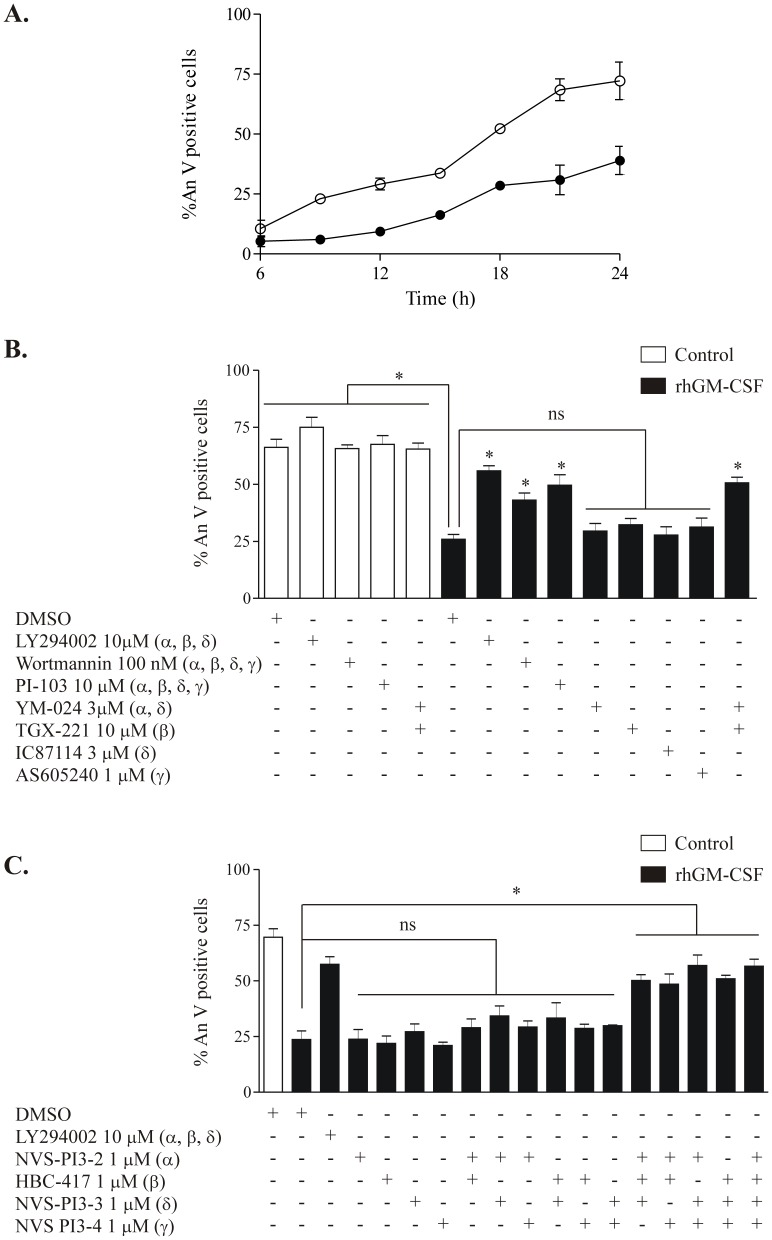
Inhibition of multiple Class I PI3K isoforms is required to abrogate the rhGM-CSF cytoprotective effect in human neutrophils. **A.** Human neutrophils were incubated in IMDM +10% autologous serum in the presence (closed symbols) or absence (open circles) of rhGM-CSF 10 ng/ml for 6–24 h. Neutrophil apoptosis was quantitated after 20 hours of culture by flow cytometry following AnV and PI staining. Data represent %AnV positive cells (PI negative plus PI positive). Data represent n = 2 independent experiment performed in triplicate. **B.** Human neutrophils (5×10^6^/ml) were pre-incubated for 20 mins in IMDM +10% autologous serum containing the indicated inhibitors or vehicle control (DMSO), prior to treatment with rhGM-CSF 10 ng/ml. Neutrophil apoptosis was quantitated after 20 hours of culture by flow cytometry following AnV and PI staining. **C.** Human neutrophils (5×10^6^/ml) were pre-incubated for 20 mins in IMDM +10% autologous serum containing the indicated inhibitors, individually or in combination or vehicle control (DMSO concentration equivalent to that applied with the combination of all four isoform-selective inhibitors). Cells were then treated with rhGM-CSF 10 ng/ml and apoptosis was quantitated after 20 hours of culture by flow cytometry following AnV and PI staining. For B and C, data represent results of three independent experiments, performed in triplicate, expressed as mean % apoptotic neutrophils ± SEM values. *p<0.01 versus GM-CSF treatment alone; ns indicates no significant difference compared with GM-CSF alone.

GM-CSF delays human neutrophil apopotosis (Figure 3A); our initial experiments confirmed that LY294002 at a concentration of 10 µM prevented rhGM-CSF-mediated human neutrophil survival without affecting the extent of constitutive neutrophil apoptosis (Figure 3B). However, whilst 10 µM LY294002 is regarded as a ‘standard’ inhibitor concentration to block Class I PI3K activity, and will effectively inhibit all three Class IA isoforms, the IC_50_ for PI3Kγ has been measured as 7.26 µM [24]. Furthermore, 10 µM LY294002 will inhibit the catalytic activity of PI3K Class IIB and III [25] as well as several non-lipid kinases including mTOR (mammalian target of rapamycin), DNA-PK (DNA-dependent protein kinase) [26] and Pim-1, whose expression is induced by GM-CSF [27] and which can phosphorylate the pro-apoptotic protein BAD leading to inhibition of apoptosis [28]. Thus we speculated that 10 µM LY294002 may not inhibit PI3Kγ fully, and may inhibit other pathways relevant to apoptosis/survival. Wortmannin (100 nM) also blocked the anti-apoptotic effect of GM-CSF, although less completely than 10 µM LY294002 (see Figure 3B); wortmannin at this concentration would be predicted to inhibit all Class I PI3Ks; however, like LY294002, it also inhibits Class IIB and III PI3Ks, and additionally is unstable in aqueous solutions, necessitating inhibitor replenishment during the 20 hour time course of this experiment.

**Figure 4:**
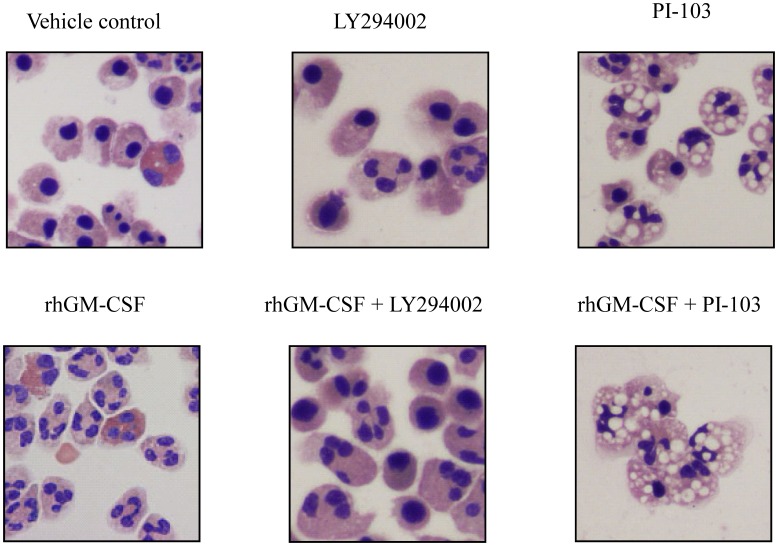
Light microscopy of human neutrophils treated with PI-103 and rhGM-CSF. Human neutrophils (5×10^6^/ml) were pre-incubated for 20 minutes in Iscove’s Modified Dulbecco’s medium (IMDM) and 10% autologous serum containing 0.1% DMSO, LY294002 (10 µM) or PI-103 (10 µM) prior to treatment with rhGM-CSF (10 ng/ml). Neutrophil apoptosis was assessed at 20 hours by morphology. Representative photomicrographs (x100) from 3 independent experiments of typical cytospins for each condition are shown. Results are representative of three independent experiments performed in triplicate.

To explore further whether the effects of LY294002 and wortmannin on GM-CSF-mediated neutrophil survival might relate to off-target effects, we used PI-103, which potently and competitively inhibits all PI3K Class I isoforms with an IC_50_ in the submicromolar range as well as DNA-PK and mTOR, but not any other of a panel of 317 other kinases [29]. Although at high concentrations (10 µM), PI-103 did indeed appear to inhibit the effects of rhGM-CSF on neutrophil apoptosis as determined by annexin V staining (Figure 3B), morphological assessment of neutrophils treated with rhGM-CSF and PI-103 did not reveal classical apoptotic morphology. Surprisingly, in cells treated with PI-103 at 1 µM or above, extensive cytoplasmic vacuolation was seen, which was not apparent in vehicle control or LY294002 treated cells (Figure 4). Although inhibitor studies using LY294002 and wortmannin predict that PI3K inhibition should result in reduced cell survival, in recent studies PI-103 did not induce apoptosis in normal CD34^+^ cells or glioma cells [30], [31]; furthermore, treatment of glioma cell lines with PI-103 led to vacuolation and LC-3 processing [32], suggesting that a form of cell death with features of autophagy rather than apoptosis may be induced in some cell types by this inhibitor. However, electron micrographs of human neutrophils treated with PI-103 demonstrated that the vacuoles although abundant were bounded by single, rather than double membranes, suggesting that this inhibitor does not induce true autophagy in human neutrophils (data not shown). In view of the unexpected effects of PI-103, we decided to explore the effect of isoform-selective PI3K inhibitors on cytokine-mediated neutrophil survival.

A selection of PI3K Class I isoform selective inhibitors were used either individually or in combination, at carefully chosen concentrations at or above their reported *in vivo* IC_50_ to inhibit PI3K α/δ (3 µM YM024), β/δ (10 µM TGX221), δ (3 µM IC87114) or γ (3 µM AS605240) (Figure 3B). Inhibition of any individual Class I isoform failed to abrogate GM-CSF mediated survival; however, the combination of 3 µM YM024 and 10 µM TGX221 (which together inhibit PI3Ks α, β and δ) blocked the survival effect to the same extent as 10 µM LY294002. To confirm these results, we used an additional independent group of isoform selective PI3K inhibitors (see Figure 1), again used at or above the IC_50_ for of the individual isoform (PI3Ks α (1 µM NVS-PI3-2), β (1 µM HBC-417; at this concentration this compound will also inhibit PI3K δ), δ (1 µM NVS-PI3-3) or γ (1 µM NVS-PI3-4); in the case of NVS-PI3-3 and NVS-PI3-4, the selected concentrations were shown to be at or above the IC_50_ for inhibitions of the TNFα-primed (NVS-PI3-3) or unprimed (NVS-PI3-4) fMLP-stimulated neutrophil oxidative burst (Figure 2). For HBC-417 and NVS-PI3-2, the selected concentrations were based on the IC_50_ values for these compounds in cell-based assays (inhibition of PKB phosphorylation in Rat1 cells transfected with the relevant PI3K isoform). In agreement with the above results, inhibition of any single Class PI3K isoform using these compounds failed to affect GM-CSF-mediated survival, as did targeting any two isoforms in combination (Figure 3C). Once again, combined inhibition of PI3K α, β and δ was equivalent to the effect observed with 10 µM LY294002; unexpectedly, inclusion of the selective PI3Kγ inhibitor NVS-PI3-4 in combination with any two other Class IA PI3K inhibitors was likewise equally effective (Figure 3C).

Importantly, none of the inhibitors used affected the rate of constitutive neutrophil apoptosis, either at 20 h or at 6 h, indicating that the cells under study were unprimed and that the compounds used even in combination were not toxic (Figure 3B and data not shown). For all inhibitor studies, the results obtained using flow cytometry were confirmed by independent morphological assessment (data not shown). These data suggest that all Class I PI3K isoforms may contribute to GM-CSF-mediated neutrophil survival, and that suppression of overall Class I PI3K activity below a certain threshold is required to abrogate this survival effect.

### Abrogation of rhGM-CSF-induced PKB Phosphorylation Requires Inhibition of Multiple PI3K Class I Isoforms

**Figure 5:**
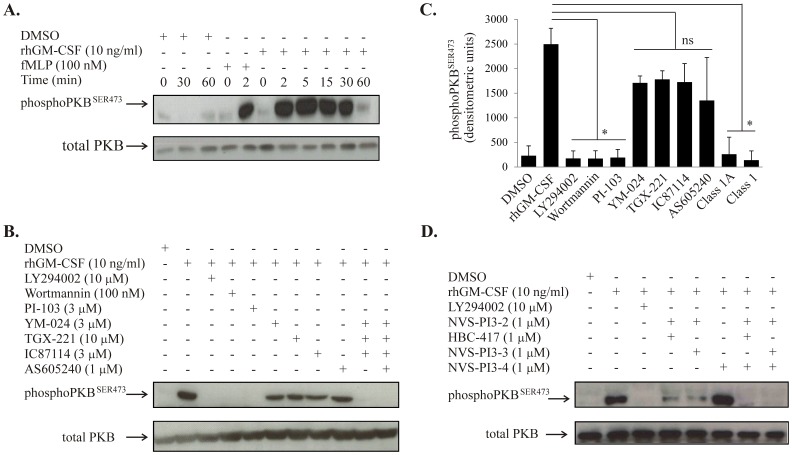
Combined inhibition of all Class IA PI3Ks abrogates rhGM-CSF mediated PKB phosphorylation. **A.** Human neutrophils (5×10^6^/ml) were incubated in IMDM +10% autologous serum and treated with rhGM-CSF (10 ng/ml) for 2, 5, 15, 30 and 60 mins or with fMLP (100 ng/ml) for 2 min as a positive control. Cells were lysed and the lysates subjected to Western blotting for total and phosphorylated PKB exactly as described. A representative immunoblot of n = 3 is shown. **B.** The effect of PI3K inhibition was determined by pre-incubating neutrophils with the indicated inhibitors for 20 minutes at 37°C prior to treatment with rhGM-CSF for 10 minutes prior to blotting for total and phosphorylated PKB. A representative immunoblot of n = 3 independent experiments is shown. **C.** Western blots from the experiments describe in B. above were scanned and analyzed using the Aida Image Analyzer 3.27 software package. Data represent mean ± SEM of 3 independent experiments. Class 1A represents YM-024 plus TGX-221 and IC87114; Class 1 as for Class 1A with the addition of AS605240. *p<0.01 versus GM-CSF alone, ns = not significantly different from GM-CSF alone. D. Human neutrophils (5×10^6^/ml) were incubated with the indicated PI3K inhibitors alone or in combination for 20 minutes at 37°C prior to treatment with rhGM-CSF for 10 minutes. Lysates were blotted for total and phosphorylated PKB as described. A representative immunoblot of n = 3 independent experiments is shown.

**Figure 6:**
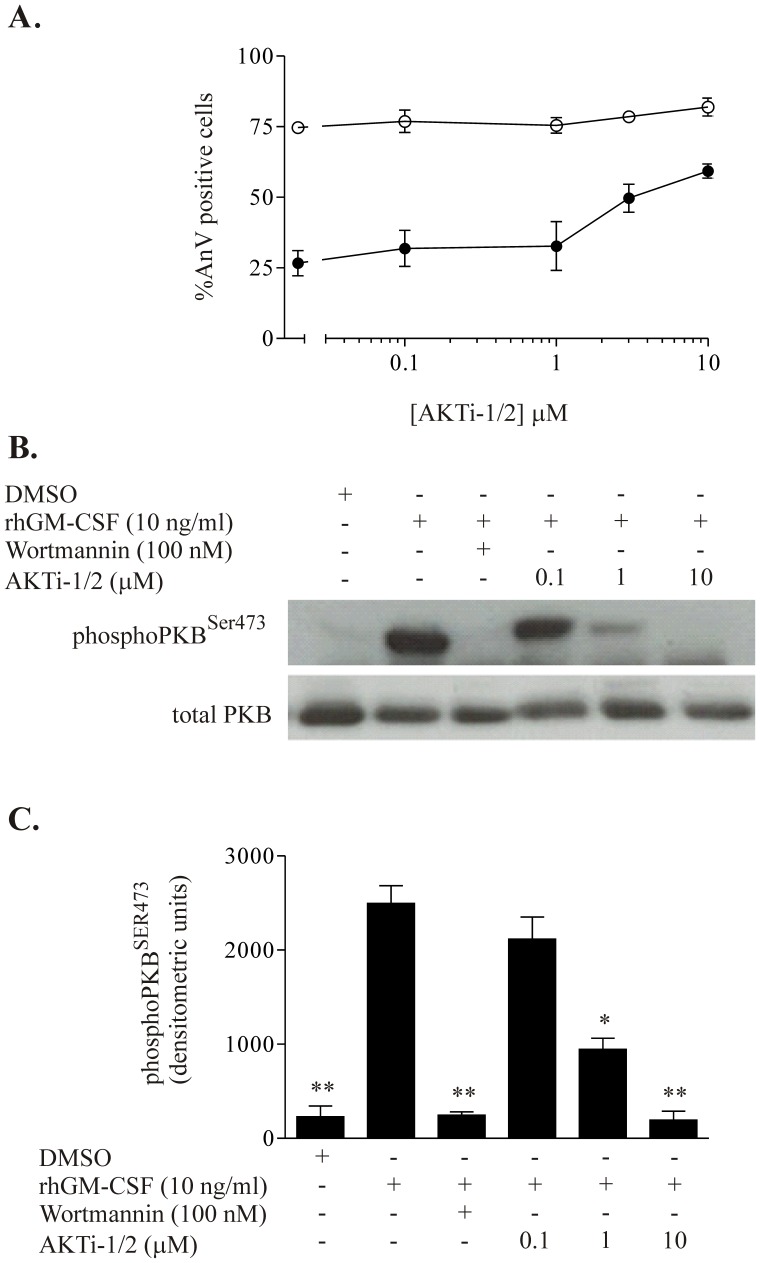
Effect of Akt inhibition on rhGM-CSF-mediated neutrophil survival and Akt^SER473^ phosphorylation. Human neutrophils (5×10^6^ cells/ml) were pre-incubated with either increasing concentrations of Akt1/2i, with 100 nm wortmannin, or with DMSO vehicle, for 20 minutes prior to treatment with 10 ng/ml rhGM-CSF or vehicle in IMDM supplemented with 10% autologous serum. **A.** Neutrophil were incubated with Akt1/2i at the indicated concentrations in the absence (open circles) or presence (closed circles) of rhGM-CSF 10 ng/ml. Apoptosis was assessed at 20 hours by morphology. * denotes *p*<0.05 for cells treated with Akt1/2i and GM-CSF versus cells treated with GM-CSF alone. **B.** Lysates from cells prepared as above were subjected to Western blotting for total and phosphorylated PKB. A representative immunoblot is shown. **C.** Densitometric analysis of n = 3 Western blots from B. *p<0.05 and **p<0.01 versus GM-CSF alone.

PKB is a downstream effector of the PI3K pathway, which has been implicated in neutrophil survival. The effect of rhGM-CSF (10 ng/ml) on the expression of cytosolic total and phosphorylated PKB was measured using Western blot analysis. PKB phosphorylation was rapid (as early as 30 seconds, data not shown) and transient (peak effect at 5 minutes with diminution to baseline by 60 minutes, Figure 5A). GM-CSF-induced PKB phosphorylation was abolished by pre-incubating the cells with broad spectrum PI3K inhibitors LY294002 or wortmannin prior to treatment with rhGM-CSF, and also by PI-103, but was not reduced significantly by any individual isoform-selective PI3K inhibitor (Figures 6B and 6C), consistent with the lack of effect of these latter compounds on neutrophil apoptosis (Figures 3A and 3B). In keeping with the combinatorial effects of these inhibitors on growth factor-mediated neutrophil survival described above, only the use of isoform-selective inhibitors together to target either Class I or Class IA PI3Ks abolished GM-CSF-mediated PKB phosphorylation (Figures 6B and 6C). In agreement with the effect of the selective PI3Kγ inhibitor HB-522 on neutrophil apoptosis, this agent alone had no effect on PKB phosphorylation but did have additive effects on this response when combined with either both α and β or α and δ inhibitors (Figure 5D). Of note, a substantial (although not complete) reduction in PKB phosphorylation was achieved by inhibition of any two isoforms (Figure 5D and data not shown) without an accompanying effect on neutrophil apoptosis. These data, together with experiments that show the PKB inhibitor AKT-i1/2 suppresses GM-CSF-mediated neutrophil survival (Figure 6) suggest that near-complete (∼85%) inhibition of GM-CSF-induced PKB phosphorylation is required to abrogate the GM-CSF mediated survival effect.

### Murine Peripheral Blood Neutrophils Undergo Time-dependent Apoptotic Cell Death, which is Delayed by rmGM-CSF

**Figure 7:**
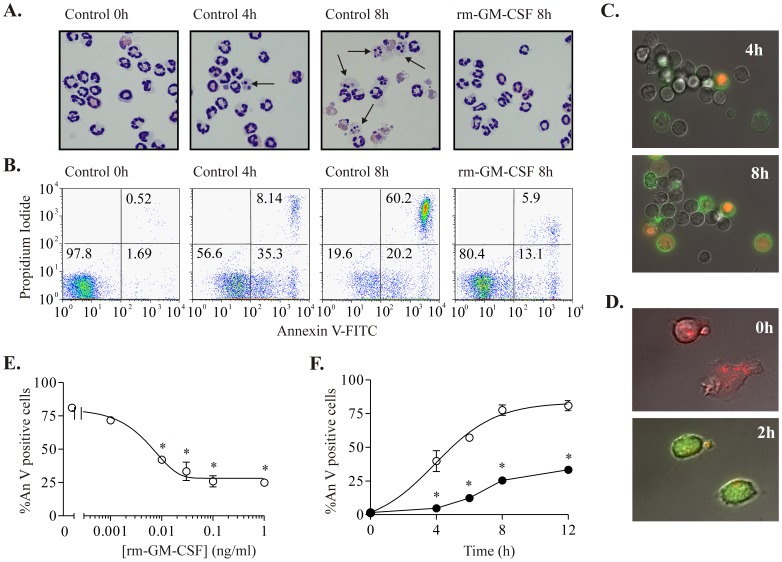
Murine peripheral blood neutrophils undergo time dependent cell death which is delayed by rmGM-CSF. Murine peripheral blood neutrophils (1×10^6^/ml) were cultured in RPMI, 10% heat inactivated FBS with 20 mM HEPES, and penicillin and streptomycin (100 U/L), and apoptosis was assessed after 0–12 hours in culture. **A.** Morphology at 0, 4 and 8 hours (vehicle control) and 8 hours (0.1 ng/ml rmGM-CSF); representative photomicrographs (x40) of cytocentrifuge preparations stained with DiffQuick™. Examples of apoptotic neutrophils are indicated by arrows. **B.** Representative flow cytometry profiles following AnV/PI staining. Neutrophils cultured for 0, 4 and 8 hour (vehicle control) and 8 hour (0.1 ng/ml rm-CSF). The percentage of neutrophils falling within each quadrant is indicated. **C.** Representative confocal images of murine peripheral blood neutrophils cultured for 4 or 8 hours and stained with An/PI as described in Methods. **D.** Representative confocal images of murine peripheral blood neutrophils incubated with JC-1 (0 or 2 hours). The dye localizes in intact mitochondria as red-shifted “J-aggregates”; loss of mitochondrial potential results in formation of JC-1 monomers and changes its emission to green. **E.** Murine peripheral blood neutrophils were incubated with vehicle or with rmGM-CSF at the concentrations indicated for 8 hour and apoptosis determined by AnV/PI staining quantified by flow cytometry (open circles). Data represent results of three independent experiments, performed in duplicate, expressed as mean % apoptotic neutrophils ± SEM. *p<0.01 compared with vehicle control-treated cells. **F.** Murine peripheral blood neutrophils were incubated with vehicle (open circles) or with rmGM-CSF 0.1 ng/ml (closed circles) for the indicated times and apoptosis was determined by AnV/PI staining quantified by flow cytometry. Data represent results of three independent experiments, performed in duplicate, expressed as mean % apoptotic neutrophils ± SEM. *p<0.01compared with control values.

The inhibitor studies described above suggest the possibility of major functional redundancy between all Class IA and IB PI3K isoforms. We wished to rule out the possibility that these results were due to off-target inhibitor effects or to poor cellular accessibility of the compounds, hence we used neutrophils from knockout mice lacking individual PI3K isoforms. To overcome the heterogeneous state of maturity of neutrophils obtained from murine bone marrow, and the variable purity of such cells (both of which variables would confound assessment of neutrophil apoptosis), we refined the previously described methodology [23] to allow consistent isolation of murine peripheral blood neutrophils from C57BL/6 wild type mice to 90±5% purity and 98% viability. We then characterized constitutive and rm-GM-CSF-modulated apoptosis in these purified mature circulating murine neutrophils cultured in RPMI supplemented with 10% FBS. Neutrophil cell death was assessed after 0, 4, 6, 8 and 12 hours in culture using two complementary techniques, standard morphological assessment (Figure 7A) and flow cytometry (Figure 7B) with annexin V-FTIC and propidium iodide staining.

Murine peripheral blood neutrophils underwent time-dependent cell death, at a rate more rapid than that observed in human cells (Figure 7). After 4–6 hours of incubation, PS exposure accelerated rapidly in control cells, with nearly 60% of neutrophils becoming annexin V positive at this time point, as measured by either flow cytometry (Figure 7B) or confocal fluorescence microscopy (Figure 7C). We also used JC-1 to study mitochondrial membrane potential in cultured murine neutrophils; within 2 hours of culture a shift from red (mitochondrial) to green (cytoplasmic) fluorescence was observed indicating collapse of the mitochondrial potential consistent with early apoptosis (Figure 7D). Murine neutrophil apoptosis was again significantly delayed by rmGM-CSF in a concentration-dependent manner at all time points studied (Figure 7E and 7F). Of note, murine neutrophils were shown to be exquisitely sensitive to rmGM-CSF with an EC_50_ of 7.5 pg/ml (Figure 7E). A concentration of 0.1 ng/ml rmGM-CSF was therefore used in all subsequent experiments, which produced a consistent and powerful cytoprotective effect.

### Functional Redundancy of Class I PI3Ks in Mediating the Cytoprotective Effect of GM-CSF in Murine Peripheral Blood Neutrophils

**Figure 8:**
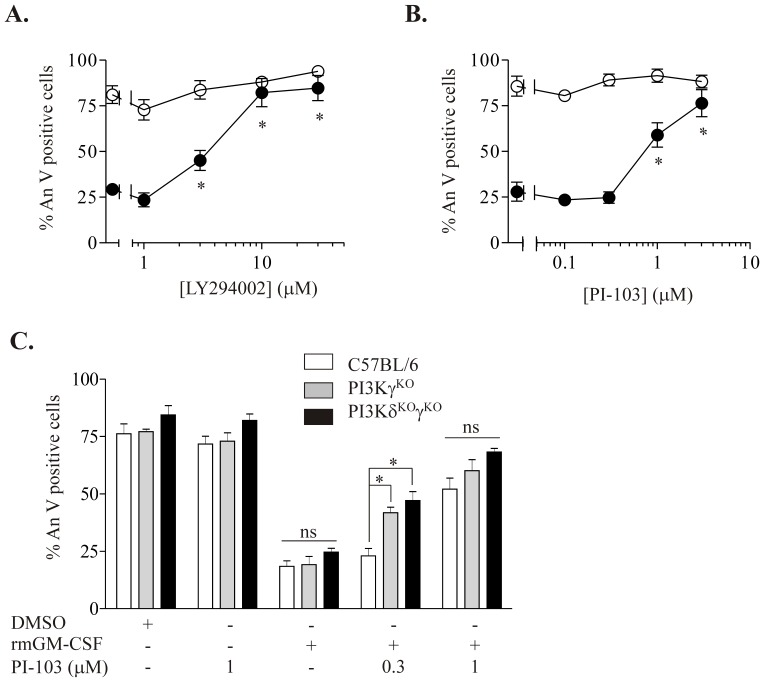
Functional redundancy of class I PI3Ks in mediating the rmGM-CSF cytoprotective effect in murine peripheral blood neutrophils. Peripheral blood neutrophils from C57BL/6 mice were pre-incubated in RPMI, 10% heat inactivated FBS with 20 mM HEPES, and penicillin and streptomycin (100 U/L) with the indicated concentrations of **A.** LY294002 and **B.** PI-103 for 30 minutes at 37°C prior to treatment with rmGM-CSF (0.1 ng/ml, closed circles) or vehicle (open circles). Apoptosis was assessed at 8 hours using flow cytometry following AnV and PI staining. Data represent results of three independent experiments, performed in duplicate, expressed as mean % apoptotic neutrophils ± SEM values. *p<0.05 for LY294002 (A.) or PI-103 (B.) versus no inhibitor. **C.** Peripheral blood neutrophils (1×10^6^/ml) from C57BL/6, p110δ^KO^γ^KO^ and p110γ^KO^ mice were pre-incubated with 0.3 and 1 µM PI-103 or vehicle prior to treatment with rmGM-CSF (0.1 ng/ml) or vehicle. Apoptosis was assessed at 8 hours using flow cytometry following AnV and PI staining. Data represent results of three independent experiments, performed in duplicate, expressed as mean % apoptotic neutrophils ± SEM. *p<0.05 for C57BL/6 versus p110δ^KO^γ^KO^ and p110γ^KO^.

The broad spectrum PI3K inhibitor LY294002 was used initially to confirm that the rmGM-CSF mediated survival of peripheral blood neutrophils from C57BL/6 mice was PI3K-dependent (Figure 8A). LY294002 abrogated the rmGM-CSF cytoprotective effect at 8 hours in a concentration-dependent manner, with a maximal (and complete) effect observed at 10 µM (Figure 8A). LY294002 had no effect on the rate of constitutive murine neutrophil apoptosis nor did it induce vacuole formation or necrosis, either at 8 h (Figure 8A) or at 4 h (data not shown). PI-103 also blocked the rmGM-CSF survival effect in a concentration-dependent manner with maximal effect achieved at 3 µM (Figure 8B); unlike human cells, murine neutrophils did not exhibit significant vacuolation on morphological assessment (data not shown) although this response was observed in the small number of contaminating monocytes within these preparations. PI-103 did not affect constitutive apoptosis at either 8 h (Figure 8B) or 4 h (data not shown).

Next, we assessed the rate of constitutive and GM-CSF-modulated apoptosis in neutrophils derived from mice lacking PI3Kγ (p110γ^KO^), or lacking both PI3Kγ and PI3Kδ (p110δ^KO^γ^KO^). As reported previously [11], significant differences were observed between peripheral white blood counts in C57B6 and p110γ^KO^ and p110δ^KO^γ^KO^ transgenic strains (Table 2). Importantly however, the rate of constitutive neutrophil apoptosis was identical in wild type and p110γ^KO^ and p110δ^KO^γ^KO^ mice; moreover, there was no difference in the magnitude of the rmGM-CSF survival response (Figure 8C). This is entirely consistent with the lack of effect of dual inhibition of PI3Kγ and PI3Kδ on GM-CSF-mediated neutrophil survival seen in human cells (Figure 3C).

We then explored whether p110γ^KO^ or p110δ^KO^γ^KO^ neutrophils display increased sensitivity to PI-103 in the presence of rmGM-CSF (Figure 8C). PI-103 0.3 µM had no effect on rmGM-CSF-induced survival of wild type neutrophils but attenuated the rmGM-CSF survival effect in neutrophils from p110γ^KO^ and p110δ^KO^γ^KO^ mice (Figure 8C). The fact that p110γ^KO^ neutrophils were equally sensitized to inhibition of Class IA isoforms as were cells which additionally lacked p110δ, provided further evidence for a role for PI3Kγ in mediating GM-CSF-induced neutrophil survival.

### The Transient Inhibition of Constitutive Neutrophil Apoptosis Observed *ex vivo* during an Exacerbation of COPD is not Sensitive to PI3K Inhibition

**Figure 9:**
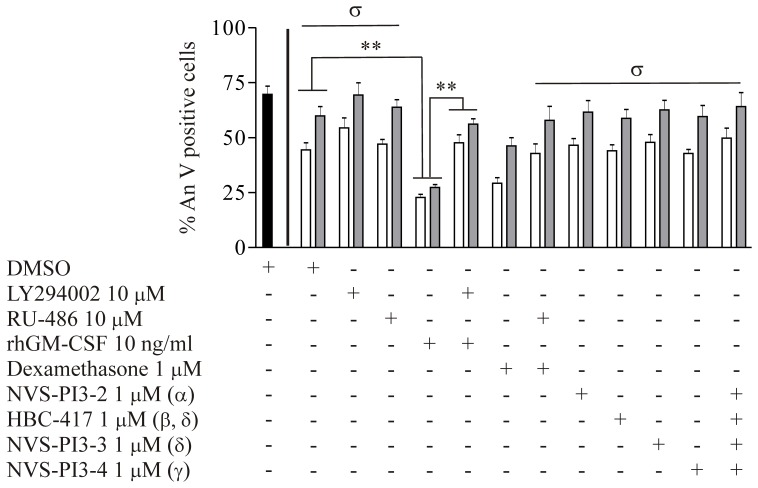
Peripheral blood neutrophils from exacerbating COPD patients display delayed apoptosis that is not sensitive to PI3K inhibition. Peripheral blood neutrophils were obtained from n = 5 patients with an exacerbation of COPD on hospital admission (open bars) and following medical treatment (grey bars) and cultured *ex vivo* in Iscove’s Modified Dulbecco’s medium (IMDM) containing 10% autologous serum. The neutrophils were pre-incubated for 20 minutes in the presence or absence of RU-486 or LY294002 or the indicated isoform-selective PI3K inhibitors prior to treatment with dexamethasone or rhGM-CSF respectively, apoptosis rates were quantitated after 20 hours of culture by flow cytometry following annexin V-FITC and propidium iodide staining. Experiments were performed in triplicate, expressed as mean % apoptotic neutrophils ± SEM values. Solid black bar indicates baseline *in vitro* neutrophil apoptosis rate for healthy human volunteers (n = 7). Similar results were obtained by morphological analysis (data not shown). *p<0.01 for cells treated with GM-CSF (or not). σ indicates that, for all conditions under the bar, p<0.01 for acute admission versus post-treatment.

Since pharmacological acceleration of neutrophil apoptosis has been shown to promote the resolution of inflammation [6], we wished to explore the relevance of PI3K signaling in a disease-based setting. Since aberrant neutrophil survival may contribute to COPD pathogenesis [33], we studied cells obtained from patients during acute exacerbations of this condition. The extent of spontaneous neutrophil apoptosis observed *ex vivo* in patients admitted to hospital with an acute exacerbation of COPD was significantly reduced in comparison to the values obtained for the same patients following 4–7 days of medical treatment (Figure 9); for comparison, the values for n = 7 healthy controls are shown in Figure 9, although we note that these cells were not isolated in parallel with the patient samples and that the healthy volunteers were not matched with the patients with regards to age and smoking habit. The lack of effect of the glucocorticoid receptor antagonist RU-486 suggested that the measured reduction in neutrophil apoptosis rates was not due to systemic corticosteroid treatment. We explored whether PI3K signals the acute pro-survival phenotype seen during COPD exacerbations and found that isoform selective PI3K inhibitors (alone or in combinations) and also LY294002 failed to restore the ability of exacerbating patient-derived neutrophils to undergo apoptosis in a timely fashion. The same cells remained fully sensitive to 10 ng/ml rhGM-CSF, which caused a fully PI3K-dependent additional survival effect (Figure 9). Thus the COPD “survival signal” for circulating neutrophils is transduced via a PI3K-independent pathway.

## Discussion

Apoptosis is a key determinant of neutrophil persistence in tissues during inflammatory disease states. We present evidence that growth factor-mediated survival of mature circulating human (and murine) neutrophils, whilst PI3K-dependent, shows complete or near-complete functional redundancy with regard to all known Class I PI3K isoforms.

Although enriched in PI3Ks δ and γ, neutrophils also express abundant amounts of PI3Kα and β. Whilst the latter isoforms play a key role in non-haemopoeitic cell survival/apoptosis, the situation in hemopoeitic cells is less clear. Class IA but not IB PI3K inhibitors were found to induce apoptosis in chronic lymphatic leukemia cells [34], whilst inhibition of PI3Kβ or PI3Kδ triggered apoptosis in acute promyelocytic leukemic cells [24]. Somewhat surprisingly, over-expression of p101, which resulted in constitutive PI3Kγ activation in T cell lines, conferred resistance to UV-induced apoptosis [35], thus there is a precedent for PI3Kγ-derived PIP_3_ to signal for enhanced immune cell survival. PI3Kγ was also implicated in a study of constitutive neutrophil apoptosis [16], but these authors did not utilize isoform-selective PI3K inhibitors or neutrophils lacking individual PI3K isoforms. Using these tools, we have shown that inhibition of PI3Kγ (and indeed other PI3K isoforms) in human neutrophils and deletion of PI3Kγ either alone or with PI3Kδ in murine neutrophils does not affect constitutive neutrophil apoptosis; additionally, neither combinations of selective inhibitors nor the non-selective Class I PI3K inhibitor LY294002 modulated basal neutrophil lifespan. Thus we have shown unequivocally that PI3K activity does not regulate constitutive neutrophil apoptosis under our conditions.

Early reports indicated that deletion of individual PI3K isoforms in mice led to complete inhibition of specific cellular functions, but subsequent studies have often revealed complex and context-dependent involvement of other isoforms, with a surprising degree of cross-talk between Class IA and Class IB enzymes. For example, mouse neutrophils lacking functional PI3Kγ exhibited profound reductions in neutrophil chemotaxis, recruitment to inflammatory foci, and respiratory burst activity [11]–[13]. However, other investigators subsequently demonstrated that, depending on the inciting stimulus, PI3Kδ also plays a significant role in neutrophil chemotactic responses [17], recruitment [18], and in the human respiratory burst when stimulated by G-protein coupled agonists [10]. More recently, it has been shown that PI3Ks β, δ and γ all play significant roles downstream of neutrophil FcγRs, with a more selective role for PI3Kβ at lower levels of stimulation [19]. PI3Kβ and δ have also been shown play redundant roles in neutrophil activation by Aspergillus hyphae [36]. Further, in murine macrophages, PI3Kβ is activated predominantly by GPCRs and is functionally redundant with PI3Kγ [37]. From this and earlier *in vitro* studies [38], [39] it is clear that there is considerable cross-talk between the Class IA and Class IB PI3K signaling pathways.

Using two separate panels of isoform-selective Class I PI3K inhibitors on human neutrophils, we have demonstrated that GM-CSF-mediated survival is unaffected by inhibition of any 2, but reversed by inhibition of any 3 of the 4 PI3K isoforms. Parallel studies confirmed a stepwise reduction in PKB phosphorylation with successive inhibition of increasing numbers of isoforms, irrespective of the precise isoform(s) targeted. These results imply that all 4 Class I PI3K isoforms are activated by ligation of the GM-CSF receptor and that they provide a common signal, PIP_3_. Further, if the main role of PIP_3_ in regulating survival is accepted to be via activation of PKB, our measurements of PKB phosphorylation suggest that only a fraction of the total PKB phosphorylated in response to 10 ng/ml GM-CSF (approximately 15%) is required to drive neutrophil longevity. This is consistent with our data indicating that near-complete abolition of PKB phosphorylation by the AKT1/2 inhibitor is required to prevent GM-CSF-mediated survival (Figure 6). However, it is also possible that PIP_3_-dependent pathways other than PKB also participate in signaling growth factor-mediated neutrophil survival.

Although we have previously demonstrated activation of PI3Kδ subsequent to, and dependent on, the activation of PI3Kγ by fMLP in human neutrophils [10], activation of PI3Kγ in response to ligation of the tyrosine kinase-linked GM-CSF receptor was unexpected; hence we performed supernatant-transfer experiments in the presence of GM-CSF neutralizing antibodies (data not shown) but found no evidence of a transferable survival factor, suggesting that activation of PI3Kγ in this setting does not operate through an autocrine loop. As discussed above, there are several lines of evidence to support the occurrence of cross-talk between PI3K Class 1A and Class 1B signaling pathways, but the precise mechanism by which this occurs downstream of the GM-CSF receptor is unclear at present and requires further investigation.

To circumvent the possibility that our interpretation of results obtained using inhibitors with human neutrophils were confused by ‘off-target’ effects, we employed neutrophils derived from the peripheral blood of transgenic mice. Although there are significant challenges in isolating these cells (the circulating mouse blood volume is only 2 ml, and neutrophils comprise only 20–30% of circulating leukocytes), the use of bone marrow neutrophils was felt to be inappropriate for these experiments as the purity and maturity of the myeloid cells obtained from this tissue is variable. We acknowledge that human and murine neutrophils may differ with regard to function and signaling [10]. Nonetheless, we found that murine peripheral blood neutrophils underwent apoptosis *ex vivo* in a manner analogous to that seen in human polymorphonuclear cells, albeit more rapidly; likewise, the response to cytokines and inhibitors was very similar to that of the corresponding human cells. Thus our finding that murine neutrophils lacking PI3Kγ alone or both PI3Kγ and PI3Kδ display normal constitutive and GM-CSF modulated apoptosis but are sensitized when compared to wild type neutrophils with regard to Class IA PI3K inhibition, supports the hypothesis that Class I PI3Ks signal GM-CSF-mediated neutrophil survival in a functionally redundant manner.

The fact that mice lacking PI3Kγ or PI3Ks γ and δ exhibit peripheral blood neutrophilia despite normal constitutive neutrophil apoptosis rates is intriguing, since circulating neutrophil numbers are tightly controlled by a range of homeostatic mechanisms. As discussed by Stark et al. [40], mice deficient in a range of leukocyte or endothelial adhesion molecules also display a peripheral blood neutrophilia. An early suggestion that this resulted simply from the inability of adhesion-deficient leukocytes to egress from the circulation (passive accumulation) was not supported by data using bone marrow chimeras [41]; instead, Stark et al. have proposed that a failure of neutrophil transmigration into the tissues results in reduced ingestion of apoptotic neutrophils by macrophages and dendritic cells, thereby failing to suppress the secretion of IL-17 which is a stimulus for granulopoeisis [40]. Whilst this hypothesis has not been tested in PI3K knockout mice, inhibition of the PI3K-Akt axis was found to inhibit macrophage production of IL-17 in a mouse model of endotoxemia [42]. Thus despite normal rates of constitutive neutrophil apoptosis, it is plausible that the failure of PI3Kγ/PI3Kγδ-deficient neutrophils to migrate to the tissues prior to undergoing local apoptosis may lead to the failure of this important homeostatic mechanism.

Our results resemble those of Foukas et al. [20], but there are a number of important differences between the two studies. Foukas et al. employed murine hemopoietic progenitor cells derived from mice lacking either PI3Kα or PI3Kδ or both, immortalized by transduction with a retrovirus encoding the proto-oncogene Hox1. In these immortalized progenitor cells, activity of any Class IA PI3K isoform was found to be sufficient to sustain proliferation or survival, although no role was demonstrated or implied for PI3Kγ. By contrast we have studied un-manipulated human and murine blood neutrophils which are terminally differentiated, non-dividing cells programmed to die by apoptosis; clearly the signaling events which determine longevity in these cells might be expected to differ from those supporting immortalized progenitor cells.

Since neutrophil apoptosis is a key factor in allowing the resolution of inflammation in a range of inflammatory diseases, pharmacological manipulation of neutrophil survival is regarded as a potential therapeutic target [6]. However, our results would suggest that even combined inhibition of PI3Kγ and δ (such dual inhibitors have been proposed as powerful potential anti-inflammatory agents [43]) would not be sufficient to modulate the survival signal imparted by a single cytokine or growth factor. Since inflammatory diseases are characterized by an array of circulating and locally generated cytokines, the prospect that selective PI3K inhibitors will be able to modulate neutrophil apoptosis in this setting seems increasingly remote. In keeping with this view, neutrophils isolated from patients hospitalized with exacerbations of COPD were extremely resistant to PI3K inhibition in terms of survival. We chose to study COPD because it is a major global cause of morbidity and mortality, and because a previous study had suggested that neutrophils from patients experiencing an exacerbation of COPD exhibit delayed apoptosis [33]. We found that circulating neutrophils from patients early in the course of disease did indeed show prolonged survival compared to cells isolated from the same individual during the recovery phase. However, the survival effect demonstrated could not be reversed by inhibition of individual PI3Ks, by combined inhibition of all four isoforms, or by LY249002 (Figure 9). During an exacerbation of COPD, and in the majority of inflammatory conditions, a wide range of circulating and tissue mediators may influence the neutrophil function and lifespan, and this is not readily reproduced *in vitro*.

In summary, we have demonstrated for the first time that there is complete functional redundancy between Class I PI3K isoforms in mediating the pro-survival signal imparted by GM-CSF to mature circulating neutrophils, and that in a human disease state characterized by delayed neutrophil apoptosis, this delay is not modulated by PI3K inhibition. This suggests that alternative strategies will be needed to modulate neutrophil apoptosis in human disease *in vivo*.
